# Field Evaluation of Temperature and Wind-Speed Sensor Performance Under Natural Icing Conditions for Power Meteorological Monitoring

**DOI:** 10.3390/s26082312

**Published:** 2026-04-09

**Authors:** Hualong Zheng, Xiaoyu Liu

**Affiliations:** Xuefeng Mountain Energy Equipment Safety National Observation and Research Station, Chongqing University, Chongqing 400044, China; 202211021032@stu.cqu.edu.cn

**Keywords:** microclimate of the power grid, ice monitoring, field observation, temperature measurement, wind speed measurement

## Abstract

Micro-meteorological monitoring systems have been widely deployed in power grids, providing essential data to support the prevention and mitigation of ice- and wind-related disasters. However, understanding of the associated error mechanisms and quantitative evaluations under freezing rain and snow remains limited, particularly in complex field environments. This study presents a field-based quantitative assessment of two key variables, air temperature and wind speed, based on comparative observations collected over multiple winter icing cycles. We analyze the coupled effects of low temperature, ice accretion, and solar radiation on temperature measurements through multi-configuration sensor comparison, and characterize the dynamic response of cup anemometers under icing conditions using cross-correlation lag analysis. Results show that temperature error is dominated by sensor installation configuration and solar radiation. Under weak solar radiation, unshielded sensors tend to record lower temperatures than a standard Stevenson screen, but once radiation exceeds 200 W/m^2^, they warm rapidly and exhibit maximum positive biases of ~8–10 °C. Ice accretion further induces a cold bias of ~1 °C and a response lag of 5–18 min, while suppressing the rapid warming driven by shortwave radiation. For wind measurements, cup anemometers show clear underestimation during ice accretion, with the error increasing nonlinearly with ice thickness to ~20% before freezing-induced failure occurs. These findings provide a basis for improved sensor deployment and interpretation of field monitoring data in cold, humid, and icing-prone environments, although the quantitative results are site-dependent.

## 1. Introduction

With the increasing impacts of global climate change and the continued expansion of long-distance clean-energy transmission, power systems are facing growing challenges in maintaining safe and stable operation under complex environments and extreme meteorological conditions. In icing-prone mountainous regions, macro-scale weather data often fail to represent the actual environment experienced by transmission lines, sensors, and monitoring devices, whereas micro-meteorological observations directly affect icing warning, equipment monitoring, and operational decision-making [1]. As a result, micro-meteorological monitoring systems have been widely deployed in power grids. However, under freezing rain, snow, and complex terrain, the accuracy and reliability of the measurements themselves remain insufficiently understood, especially for air temperature and wind speed, which are two key variables for icing diagnosis and mitigation.

Previous studies have shown that air-temperature measurements are strongly affected by shielding, ventilation, radiation loading, and sensor response characteristics [2]. In naturally ventilated screens or exposed field deployments, insufficient airflow enhances radiation-induced bias, while finite sensor response damps rapid temperature changes and distorts extremes [3,4]. Harrison and Burt showed that naturally ventilated thermometer screens are generally reliable but may exhibit substantial uncertainty in light winds because of combined radiation-exchange and response-lag effects [5], whereas Burt and de Podesta demonstrated that response time depends strongly on probe diameter and airflow, and that most commercial probes do not satisfy WMO guidance under ventilation conditions typical of passively ventilated screens [6,7]. Recent studies have further shown that direct and reflected radiation, airflow organization, shield structure, and surface reflectivity jointly control temperature-measurement performance, while optimized naturally ventilated designs combined with CFD [8] or model-assisted correction [9,10,11] can substantially reduce radiation error under controlled conditions. These issues become more critical over snow- and ice-covered surfaces, Huwald et al. identified surface albedo as a major control on radiative error magnitude and showed that reflected shortwave radiation may be even more influential than incident radiation [12], Musacchio et al. demonstrated that none of the tested instruments were immune to warming caused by snow-reflected radiation, although forced ventilation could partly reduce the effect [13], and Arck and Scherer showed that naturally ventilated sensors over snow may experience both instantaneous radiation error and delayed error propagation because of slow response time [14]. These findings are directly relevant to icing environments, where snow cover, ice accretion, and complex topography jointly reshape the local radiative environment and complicate the interpretation of temperature bias. Compared with temperature measurements, wind-speed measurements under icing have often been discussed mainly in terms of complete mechanical failure, yet recent studies indicate that degradation begins well before stoppage. Cup-anemometer performance can deteriorate progressively because of contamination, air-density variation, aerodynamic disturbance, bearing drift, and icing-related surface modification. Kimura et al. showed that aerodynamic penalties depend more on ice morphology than on ice thickness alone, with dry-growth ice causing much larger degradation than wet-growth ice [15]. Alfonso-Corcuera et al. reported that ice or dirt accumulation inside the cups can already cause appreciable wind-speed underestimation, depending on both the amount and distribution of accretion [16], while Fortin et al. showed that both heated and unheated cup anemometers experience performance loss during icing exposure and that heating does not necessarily ensure accurate measurement under severe conditions [17]. In addition, air-density variation changes the balance between aerodynamic torque and friction torque [18], while ageing-related drift can also introduce systematic underestimation [19].

Complex terrain further complicates sensor performance in icing-prone mountain environments. By modifying local airflow, radiation exchange, and moisture distribution, terrain affects both icing development and measurement exposure conditions [20]. Field studies have shown that mountain wind profiles differ substantially from those over flat terrain, and that valley and canyon winds cannot be described reliably by conventional assumptions [21]. Snow-covered terrain also reshapes local energy balance through slope-modulated shortwave radiation, surrounding-terrain longwave re-illumination, and elevation-dependent temperature gradients [22]. In addition, icing-frequency mapping indicates that local terrain can cause large site-to-site deviations even where regional patterns are captured reasonably well [23]. Recent reviews likewise emphasize that passes, windward slopes, valleys, lakes, and cloud-immersed mountain flanks are especially favorable for transmission-line icing.

The above issues are closely related to established observational standards. The WMO Guide to Instruments and Methods of Observation and related AWS specifications emphasize the importance of siting, exposure, shielding, ventilation, calibration traceability, maintenance, metadata, and uncertainty analysis for reliable surface meteorological observations [24,25]. At the same time, studies on airflow within Stevenson screens show that internal ventilation is often much weaker than commonly assumed, which increases both radiation-related uncertainty and sensor response time [26]. Therefore, any field-based assessment of meteorological sensor performance under icing should be grounded in a clear reference basis, calibration statement, and uncertainty framework.

Despite the growing body of work on radiation-induced temperature bias, response-time limitations, cup-anemometer degradation, and the influence of snow and topography, two important gaps remain. First, most studies on meteorological sensor performance have been conducted under routine exposure conditions, laboratory settings, wind-tunnel experiments, or simplified evaluation frameworks. Second, studies in natural icing and cold-region environments have more often focused on icing parameters, ice loads, or surface energy balance than on the performance of the meteorological sensors themselves [27,28,29,30]. Consequently, field-based evidence remains limited on how installation configuration, radiative forcing, icing-induced thermal inertia, and wind-speed degradation jointly affect temperature and wind observations under natural icing in complex mountain environments [31,32]. This gap is particularly important for power-grid meteorological monitoring, where both terrain and icing processes may substantially alter sensor performance and data interpretation.

To address this gap, this study investigates how natural icing modifies the static and dynamic measurement performance of air temperature and wind speed under realistic power-grid monitoring conditions. Field observations were conducted at the Xuefeng Mountain Energy Equipment Safety National Observation and Research Station during the 2025 natural icing season. To clarify the scientific focus of the study, the following research questions are addressed:

1. How do shielding, ventilation, solar radiation, and icing jointly affect air-temperature measurement bias under different installation configurations?
2. How does ice accretion alter the temporal response of air-temperature sensors under field conditions?
3. How does natural icing degrade the measurement performance of cup anemometers and lead to freezing-induced failure?

The main contribution of this work is a field-based quantitative evaluation of both temperature- and wind-speed-sensor performance under natural icing conditions, integrating three aspects in a single observational framework:

1. Quantification of the effects of shielding, ventilation, solar radiation, and icing on temperature-measurement bias under different installation configurations.
2. Characterization of icing-induced response lag of temperature sensors using cross-correlation analysis interpreted in terms of thermal inertia and transient heat transfer.
3. Evaluation of cup-anemometer wind-speed degradation through a field de-icing comparison experiment using co-located cup anemometers.

The novelty of this study lies in its field-based assessment under realistic icing and cold-mountain conditions, rather than laboratory or idealized experiments, providing both site-specific evidence and practical implications for sensor deployment and data interpretation in icing-prone mountainous environments and power-grid monitoring systems.

## 2. Experimental Observations

### 2.1. Experimental Conditions

Field observations were conducted at the Xuefeng Mountain National Field Scientific Observation and Research Station for Energy Equipment Safety, located in the Xuefeng Mountain National Forest Park, Hunan Province, China (110.4083° E, 27.3125° N), at an elevation of approximately 1500 m. The station lies in the middle section of the main Xuefeng Mountain ridge and is characterized by typical high-mountain valley terrain. The Xuefeng Mountain region is an important quasi-stationary frontal activity zone in China, where wintertime interactions between cold air and warm, moist southwesterly airflow frequently occur. Distinct terrain-induced flow acceleration develops in local mountain passes and valleys, with wind speeds that may exceed the incoming flow by more than 1.5 times. Such acceleration not only increases the impact kinetic energy of supercooled droplets, but also promotes local moisture convergence, thereby favoring frequent and pronounced icing. The experimental site is located near a summit saddle with little topographic obstruction and is therefore highly exposed to both airflow and solar irradiance. The underlying surface is mainly covered by rough secondary natural forest, and the surrounding gullies are dense and comb-like in distribution, which favors local microscale circulation, moisture retention, and repeated droplet impingement on exposed structures. As a result, the site exhibits a high icing risk, with an average of about 100 icing days per year, more than 200 high-humidity days, annual precipitation of up to 1800 mm, and a maximum recorded icing thickness of about 500 mm. These topographic and climatic features make the site representative of highly exposed icing-prone mountain stations for power-grid meteorological monitoring in southern China. A standard surface meteorological station is installed at the site, and the temperature observation in the Stevenson screen complies with ISO 19289 [33], providing a standardized practical reference for intercomparison in this study.

**Figure 1:**
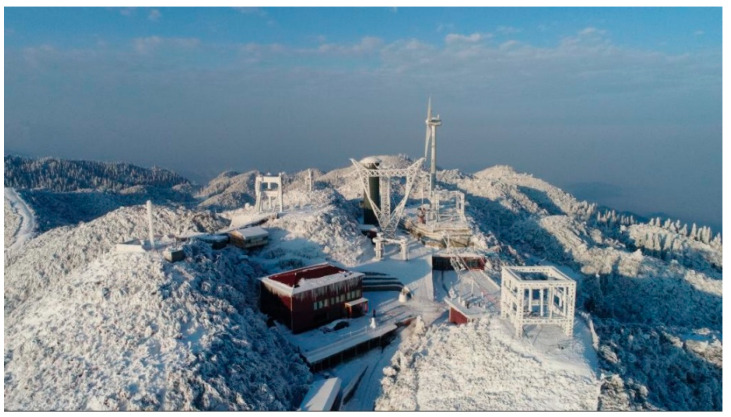
Panoramic view of the Xuefeng Mountain field observation station, showing the high-elevation mountainous environment and the general exposure conditions of the icing observation site.

From January to March 2025, multiple meteorological sensors were deployed within the station’s icing observation site and operated in conjunction with existing platforms (e.g., the on-site meteorological station) to investigate how sensor measurement errors vary under different solar radiation levels, wind-speed conditions, and icing stages. Figure 1 shows a panoramic view of the Xuefeng Mountain field observation station.

### 2.2. Experimental Setup

The investigated station is located in a complex mountainous icing-prone area and represents a typical monitoring node within the regional power meteorological observation network, where winter icing frequently affects sensor exposure and data reliability. The surrounding topography can strongly modulate local airflow, radiation exposure, moisture transport, and icing development, thereby influencing both environmental forcing and sensor measurement conditions. Therefore, the experimental design considered not only the sensor configuration itself, but also the topographic setting of the site, so that the observed measurement characteristics could be interpreted in the context of practical monitoring in complex mountainous terrain.

For temperature measurements, two differentiated comparative observation schemes were designed. First, under non-icing conditions, sensor responses under different shielding configurations were compared to clarify the respective roles of radiation and convection under typical winter low-temperature conditions. During icing events, the three sensors experienced different icing severities, enabling a direct assessment of how ice accretion affects temperature measurements. Using the sensor inside the Stevenson screen as an approximate reference, we quantified the temperature bias, response lag, and abrupt-change features of the other two sensors.

**Figure 2:**
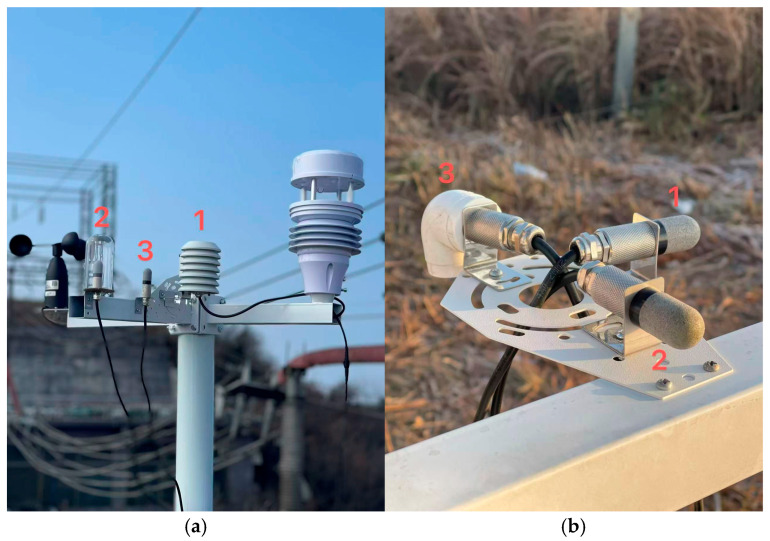
Deployment configurations of the three temperature–humidity sensors under different exposure conditions. (**a**) Sensor arrangement before the icing period, used to compare the effects of shielding and ventilation under non-icing winter conditions. (**b**) Sensor arrangement during the icing period, designed to produce different icing exposures for Sensors 1, 2, and 3 under the same field environment.

The instruments used in Figure 2 include three SHT35 temperature–humidity sensors, two three-cup anemometers, and one four-transducer ultrasonic anemometer. Table 1 shows the sensor deployment configuration. To facilitate comparison, the main specifications of the sensors used in this study are summarized in Table 2.

To assess the degradation of cup-anemometer measurements under natural icing conditions, two identical cup anemometers were installed at the same site and at the same height for synchronous observation. During representative icing periods, one instrument was manually de-iced at intervals, whereas the other was kept under natural icing conditions. The measurement difference between the de-iced and non-de-iced anemometers was then used to evaluate the impact of ice accretion on wind-speed observation. A co-located ultrasonic anemometer was additionally used to confirm the presence of ambient wind when the cup anemometer showed persistent near-zero output, so as to verify that such periods corresponded to freezing conditions rather than actual calm winds.

### 2.3. Calibration and Reference Basis

Before field deployment, all sensors were subjected to routine pre-deployment checks, including power-on stabilization, verification of synchronized data logging, inspection of communication status, and basic consistency checks under the same ambient conditions. These procedures were intended to confirm normal instrument operation and to minimize non-environmental discrepancies caused by acquisition or synchronization problems. Owing to the practical constraints of long-term field observations under natural icing conditions, no full laboratory recalibration over the entire measurement range was performed for all sensors prior to deployment. In this study, the manufacturer-provided calibration was adopted as the baseline metrological reference, and a practical basis for sensor comparability was established through unified data acquisition and co-located deployment under consistent temporal and broadly comparable exposure conditions. Accordingly, the present assessment is operational and comparative in nature. As the main objective was to quantify measurement bias and performance degradation under natural icing, the reported results should be interpreted primarily as relative field differences rather than absolute sensor errors in a strict metrological sense.

For temperature analysis, the Stevenson-screen observation from the on-site standard meteorological station was used as a practical reference for intercomparison. This choice was motivated not only by its compliance with standardized shielding and exposure requirements, but also by the Stevenson screen’s well-established design, which reduces direct radiative heating and achieves more representative thermal coupling with the surrounding air than unshielded or weakly ventilated sensor setups. In addition, because the reference station and the experimental sensors were located within the same observation area, the comparative measurements were obtained under broadly comparable large-scale meteorological conditions. Therefore, the Stevenson-screen temperature was considered an appropriate standardized working reference for evaluating relative measurement bias among different installation configurations. Nevertheless, it should not be interpreted as an absolute true value, because naturally ventilated screen observations may still be influenced by low wind speed, local radiation conditions, and other environmental factors. Accordingly, the reported temperature biases in this study should be understood as field-comparative differences relative to a standardized reference environment rather than as absolute calibration errors.

### 2.4. Uncertainty Analysis

The uncertainty in the present study arises mainly from sensor accuracy, reference measurements, temporal resolution, and data-processing choices. The nominal uncertainties of the temperature–humidity sensors, cup anemometers, and ultrasonic anemometer were taken from the manufacturer’s specifications and factory calibration information (Table 2). For temperature analysis, the Stevenson-screen observation was used as a standardized practical reference rather than an absolute true value. Accordingly, the reported temperature biases should be interpreted as field-comparative differences under broadly comparable exposure conditions. For wind analysis, the co-located ultrasonic anemometer was used as a working reference in the evaluation of cup-anemometer performance; however, its own potential limitations under icing conditions were also recognized. Accordingly, the present uncertainty analysis focuses on identifying the main sources of uncertainty under field conditions rather than performing a full metrological uncertainty propagation.

Additional uncertainty is associated with data processing and methodological choices. In particular, the lag estimation based on cross-correlation is constrained by the temporal resolution of the data and may also be affected by the selection of smoothing parameters and lag-search ranges. In the present study, these processing settings were chosen to balance short-term fluctuation suppression and retention of the dominant temperature-evolution signal. For icing-stage analysis, uncertainty may also arise from the determination of icing thickness, the identification of relatively stable periods, and the division of observational stages. These factors mainly affect the exact numerical values of short-term estimates, but they do not alter the overall temporal evolution or the comparative trends discussed in this study.

It should also be noted that differences among sensors may reflect not only intrinsic instrument performance but also the influence of local exposure conditions, which is itself part of the research objective in this study. Therefore, the present uncertainty analysis does not seek to remove all environmental heterogeneity, but rather to distinguish meaningful field-observed differences from discrepancies that are comparable to the nominal instrument uncertainty. In general, when the observed bias or lag was substantially larger than the nominal sensor uncertainty and remained consistent under reasonable data-processing settings, the result was considered to have practical physical significance. By contrast, small differences close to the instrument uncertainty level were interpreted with caution. It should also be noted that the present uncertainty treatment is primarily qualitative or semi-quantitative, and is intended to identify the main sources and practical implications of uncertainty rather than to establish a complete quantitative uncertainty budget.

### 2.5. Data Processing and Analysis Methods

All sensor data were first organized according to their timestamps and then aligned onto a unified time base for comparative analysis. Measurements from different instruments were synchronized under the same acquisition framework to ensure temporal consistency among temperature, wind-speed, and radiation records. Subsequent analyses were performed on time-matched datasets so that inter-sensor differences could be interpreted under the same meteorological background.

Prior to statistical analysis, the raw data were subjected to quality control to remove physically unrealistic values and sensor-failure periods. Temperature values outside the range of −30 °C to 40 °C were treated as invalid and excluded from further analysis. For the cup anemometer, a “frozen” state was identified when the recorded wind speed remained 0 for five consecutive minutes while the co-located ultrasonic wind speed exceeded 1 m/s; data from these periods were excluded from the error wind-speed statistics. For de-icing experiments, short time intervals immediately affected by external thermal disturbance were also excluded in order to avoid direct interference from the de-icing process itself.

Temperature bias was defined as the difference between the measured temperature of each sensor and the Stevenson-screen temperature used here as a standardized practical reference at the same time. Because no internationally unified classification scheme exists for near-surface shortwave radiation in this type of application, four radiation levels were defined according to the typical clear-sky global radiation range (0–1200 W/m^2^) and the relative strength of radiation-induced heating on temperature sensors: weak (0–200 W/m^2^), moderate (200–600 W/m^2^), strong (600–1000 W/m^2^), and very strong (>1000 W/m^2^). This classification was used for grouped statistical analysis of temperature-measurement bias under different radiative forcing conditions. Descriptive statistics, including mean value, median, and percentile range, were used to characterize the distribution of temperature errors under different exposure configurations and radiation levels.

To quantify the icing-induced delay in temperature response, cross-correlation analysis was applied to paired temperature series after temporal alignment. The lag corresponding to the maximum cross-correlation coefficient was taken as the characteristic response delay between sensors. Where necessary, smoothing was applied before lag estimation to suppress short-period noise while retaining the dominant temperature-evolution signal. The lag results were interpreted at the minute scale, consistent with the temporal resolution of the synchronized datasets.

For wind-speed analysis, the degradation of wind-speed measurement performance during icing was evaluated through a de-icing comparison experiment using two co-located cup anemometers of the same model, one of which was subjected to de-icing treatment. Representative icing periods were selected according to data continuity, observable icing development, and the availability of comparable records from both instruments. For analyses involving icing stages or ice-thickness intervals, the data were grouped according to the corresponding field observations in order to characterize the evolution of cup-anemometer performance during ice accretion.

Overall, the above procedures provided a unified data-processing basis for the subsequent analyses of temperature bias under different installation configurations, icing-induced response lag, and wind-speed measurement degradation under natural icing conditions.

## 3. Results and Discussion

**Figure 3:**
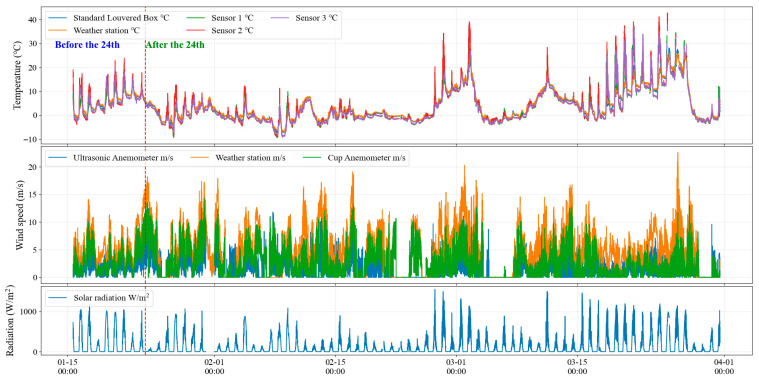
Time series of the synchronized meteorological observations during the study period, including temperature, wind speed, and radiation records used for the subsequent comparative analyses.

Figure 3 shows the trends in various meteorological data throughout the observation period.

### 3.1. The Effect of Arrangement on Temperature Measurement

**Figure 4:**
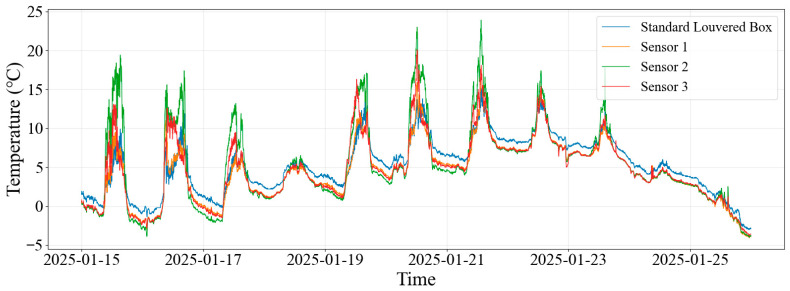
Time series of air temperature measured by the three SHT35 sensors from 15 to 25 January 2025, together with the Stevenson-screen reference temperature, showing the effects of different installation configurations under non-icing conditions.

Figure 4 shows the actual measurement data from three SHT35 sensors during the period from 15 to 24 January.

Overall, sensors exposed to solar radiation exhibited pronounced daytime warming, reaching peak temperatures around midday when solar irradiance was highest, and then cooling rapidly as irradiance decreased. In contrast, the temperature measured in the standard Stevenson screen remained relatively stable and served as a baseline reference. Sensor 1 tracked the reference most closely, with a slightly higher daytime peak and only minor nighttime differences. Sensor 2 showed a substantially higher daytime peak, indicating a stronger radiation-induced warming response. Sensor 3 was often lower at night but higher during the day, resulting in the most pronounced diurnal contrast. In general, the influence of installation configuration on temperature was mainly reflected in the amplification of daytime warming peaks and differences in nocturnal cooling, highlighting the strong effects of ventilation, exposure, and solar radiation on transient temperature response.

**Figure 5:**
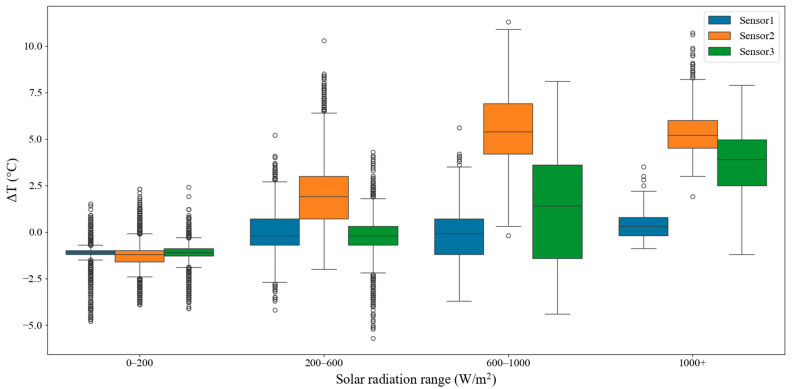
Boxplots of temperature bias relative to the Stevenson-screen reference under four solar-radiation intervals (0–200, 200–600, 600–1000, and >1000 W/m^2^) for the three sensor configurations.

Solar radiation was discretized into four intervals to further compare temperature differences across radiation levels (Figure 5). Under weak radiation (0–200 W/m^2^), all three deployment configurations exhibited a stable negative bias, with temperature errors clustering around −1 °C. Because the sample size in this interval was much larger than in the other radiation categories—and because temperature during this stage is jointly influenced by wind speed, humidity, nocturnal radiative cooling, and local inversions—the error dispersion was greater, yielding the densest outliers in the boxplots.

As radiation increased to 200–600 W/m^2^, Temperature 2 (non-ventilated and directly irradiated) developed a clear positive bias, with a median of approximately +1.8 °C, whereas Temperature 1 and Temperature 3 remained fluctuating around 0 °C. In the 600–1000 W/m^2^ interval, the positive bias of Temperature 2 expanded rapidly, with an interquartile range of roughly 4–7 °C and maxima exceeding 10 °C, indicating substantial heat accumulation within the non-ventilated enclosure under moderate-to-high radiation. In contrast, Temperature 3, which was directly exposed but naturally ventilated, remained constrained by wind-driven cooling; however, its error spread increased noticeably to within about ±1 °C, while Temperature 1 (shielded and ventilated) consistently maintained the smallest errors. Under very strong radiation (>1000 W/m^2^), Temperature 2 exhibited a tendency toward thermal saturation, with a median of ~+5.3 °C and an interquartile range of 4.2–6.2 °C, and extreme values exceeding +11 °C. The error of Temperature 3 also intensified, with the median rising to ~3 °C. By contrast, Temperature 1 remained stable and close to 0 °C even under very strong radiation.

**Figure 6:**
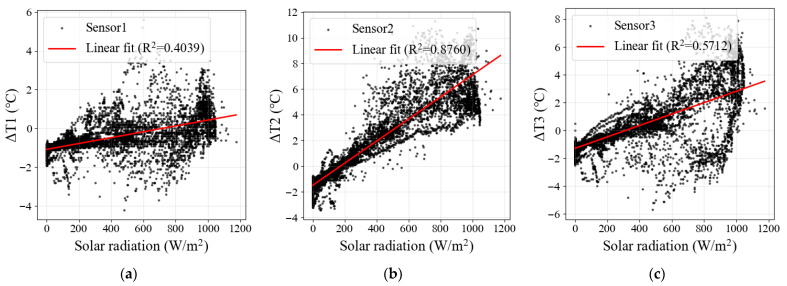
Scatterplots of temperature bias relative to the Stevenson-screen reference as a function of solar radiation for the three sensor configurations: (**a**) Sensor 1, (**b**) Sensor 2, and (**c**) Sensor 3. Red lines indicate linear regression fits, highlighting the different radiation sensitivities under the three installation conditions.

Figure 6 shows the dependence of temperature error on solar radiation for the three sensor configurations. A clear positive trend was observed for all sensors, but the response intensity differed substantially among configurations. Sensor 2 exhibited the steepest increase and the largest positive deviation under high-radiation conditions, whereas Sensor 1 remained comparatively stable and close to the reference. Sensor 3 showed an intermediate response, reflecting the combined influence of direct exposure and natural ventilation.

To quantify these differences, linear regression models were fitted between temperature error and solar radiation, and HC3 robust standard errors were applied to account for heteroscedasticity. As summarized in Table 3, the slopes for Sensors 1, 2, and 3 were 0.001511, 0.008597, and 0.004103 °C/(W·m^−2^), respectively, equivalent to error increases of 0.151, 0.860, and 0.410 °C per 100 W/m^2^ increase in solar radiation. All regression slopes were highly significant (*p* < 0.001), and the 95% confidence intervals remained entirely positive. The fitted R^2^ values were 0.4039, 0.8760, and 0.5712 for Sensors 1, 2, and 3, respectively, indicating that solar radiation explained most of the error variation for Sensor 2, but a smaller proportion for Sensors 1 and 3.

**Figure 7:**
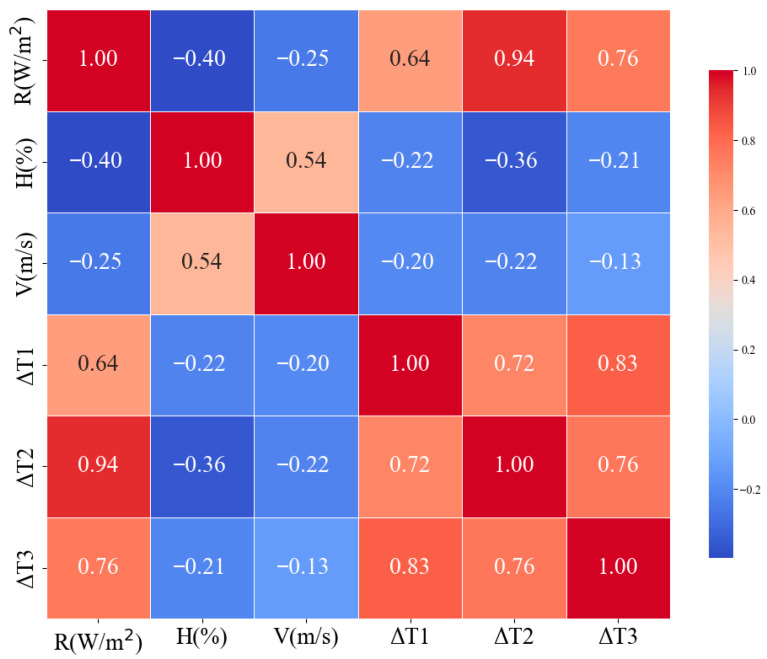
Correlation heatmap showing the relationships between temperature bias and the main meteorological variables for the three sensor configurations.

This ranking is consistent with the correlation heatmap in Figure 7, where the correlation coefficients between temperature error and solar radiation increased from 0.64 for Sensor 1 to 0.76 for Sensor 3 and 0.94 for Sensor 2. These results suggest that the deterioration in shielding and ventilation conditions is associated with a stronger first-order radiation dependence of temperature-measurement error. It should be noted that the present regression analysis is intended primarily to provide a first-order quantitative description of the relationship between solar radiation and temperature error. Because the models are univariate, they do not explicitly account for possible temporal covariation with wind speed, humidity, or other environmental variables. The results should therefore be interpreted cautiously as simplified empirical relationships under field conditions. From an energy-balance perspective [34], the temperature error can be conceptually interpreted as the result of competing radiative and non-radiative heat exchanges around the sensing element:

(1) $$Q_{net}=Q_{sw}+Q_{lw}-Q_{conv}-Q_{cond}$$

where $Q_{sw}$ is absorbed shortwave radiation, $Q_{lw}$ is longwave radiative exchange, $Q_{conv}$ is convective heat loss, and $Q_{cond}$ is conductive heat transfer through the housing and support structure. Under outdoor exposure, insufficient shielding likely increases radiative heat gain, whereas weak ventilation may suppress convective heat dissipation, thereby favoring heat accumulation around the sensor. Considering the installation configurations, the dominant factors affecting temperature-measurement performance can be further clarified within this energy-balance framework.

Considering the installation configurations, the dominant factors affecting temperature-measurement performance can be further clarified within an energy-balance framework. Comparing Sensors 1 and 3, Sensor 3 exhibits a markedly larger positive shift and greater dispersion than Sensor 1 under the same radiation levels, indicating that effective shielding can substantially reduce radiative heat gain caused by shortwave absorption and heat conduction through the housing, thereby improving measurement stability.

Comparing Sensors 2 and 3, Sensor 2 shows a stronger and more consistent positive shift under high radiation, indicating that insufficient ventilation weakens convective heat dissipation and promotes heat accumulation inside the protective structure, thereby amplifying radiation-induced errors. In contrast, although Sensor 3 partially offsets radiative heating through natural ventilation, its error distribution is more sensitive to wind-speed variability, resulting in greater dispersion.

In summary, the observed differences among the three configurations can be interpreted as the result of different balances between radiative heating and convective cooling. Shielding mainly controls the magnitude of radiative heat input, whereas ventilation primarily regulates heat dissipation and error dispersion. Therefore, the combination of shielding and ventilation is essential for suppressing radiation-induced warming bias and improving temperature-measurement stability in outdoor observations.

To further distinguish the diurnal characteristics of temperature errors under different radiative environments, clear and cloudy days were classified using the clearness index(k_cs_). In meteorological studies, cloud conditions are commonly identified using parameters such as cloud cover, radiation intensity, and atmospheric transmittance [35]. A widely used approach is to compare measured global radiation with clear-sky radiation, thereby quantifying cloud effects on radiative transfer [36].

In this study, the clearness index is defined as:

(2) $$k_{cs}\left(t\right)=\frac{GHI_{obs}\left(t\right)}{GHI_{clear}\left(t\right)}$$

where $GHI_{obs}\left(t\right)$ denotes the measured global horizontal irradiance and $GHI_{clear}\left(t\right)$ is the clear-sky irradiance estimated using a clear-sky model. The following thresholds were adopted for classification: clear-sky conditions when $k_{cs}\left(t\right)$ > 0.75, and cloudy conditions when $k_{cs}\left(t\right)$ < 0.3. Based on the calculated index, 14, 15, 16, 17, 19, 20, 21, and 23 January were classified as clear days, while 18, 22, and 24 January were classified as cloudy days.

**Figure 8:**
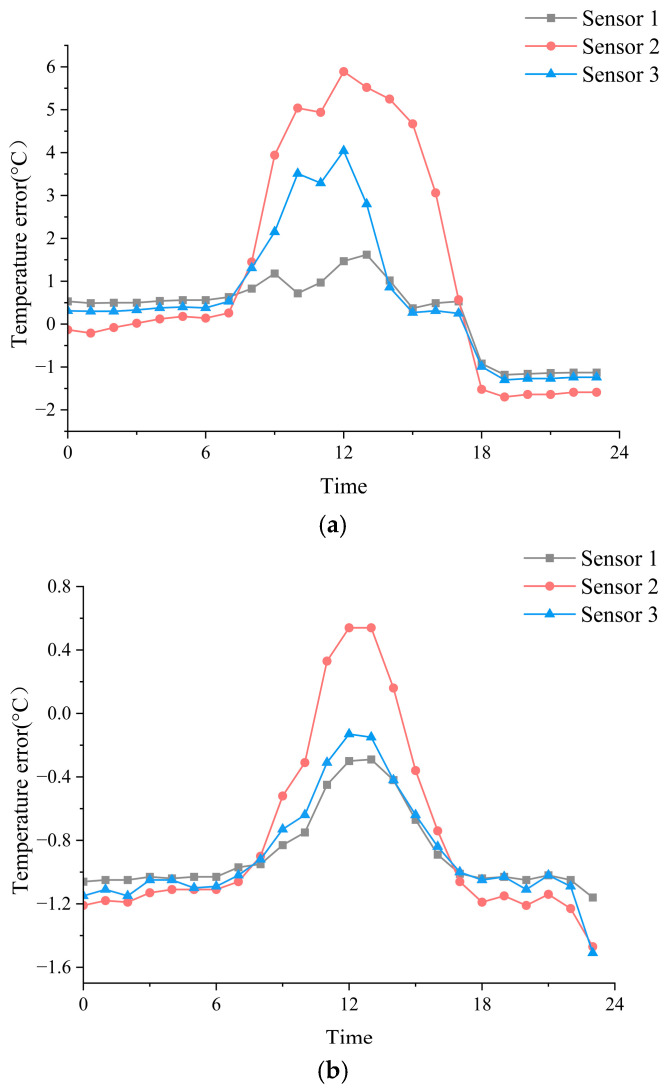
Mean diurnal cycles of temperature bias relative to the Stevenson-screen reference under (**a**) clear-sky and (**b**) cloudy conditions for the three sensor configurations.

Figure 8 shows the diurnal cycles of temperature errors for the three sensor configurations under clear and cloudy conditions. Under clear skies, all non-standard installations exhibited pronounced daytime positive biases, with error peaks occurring around the near-noon period. Compared with cloudy conditions, the daytime error amplitude was substantially larger, indicating that shortwave radiative heating dominated error formation during periods of strong insolation. Sensor 2 showed the strongest daytime amplification, followed by Sensor 3, whereas Sensor 1 remained comparatively stable. In addition, the maximum error occurred slightly later than the radiation peak, suggesting a non-negligible thermal inertia associated with the sensor housing and surrounding structure.

In contrast, under cloudy conditions, the diurnal amplitude of temperature error was markedly reduced, and all configurations were dominated by persistent negative biases for most of the day [37]. This indicates that when shortwave radiation is strongly attenuated by cloud cover, radiation-induced warming is suppressed and the controlling mechanism shifts away from a radiation-dominated regime. In this case, the net thermal balance of the sensor becomes more strongly influenced by ventilation efficiency, structural heat exchange, and local microenvironmental differences, resulting in smaller diurnal amplitudes and weaker daytime positive deviations. These results suggest that the diurnal structure of temperature error is governed not only by radiative heating, but also by the balance between radiative heat gain, convective cooling, and structural heat storage or release.

Therefore, the Stevenson screen should be regarded as a standardized reference environment rather than a perfect representation of undisturbed ambient air temperature under all microenvironmental conditions. The observed deviations mainly reflect differences in how various sensor structures respond to radiative forcing and convective heat exchange, rather than measurement error alone. From an energy-balance perspective, insufficient shielding increases shortwave radiative heat gain, whereas weak ventilation suppresses convective heat dissipation; together, these processes promote heat accumulation around the sensing element and amplify the positive temperature bias under clear-sky conditions. By contrast, under weak-radiation or cloudy conditions, structural ventilation and heat-exchange characteristics become relatively more important.

### 3.2. The Impact of Icing on Temperature Sensor Measurements

**Figure 9:**
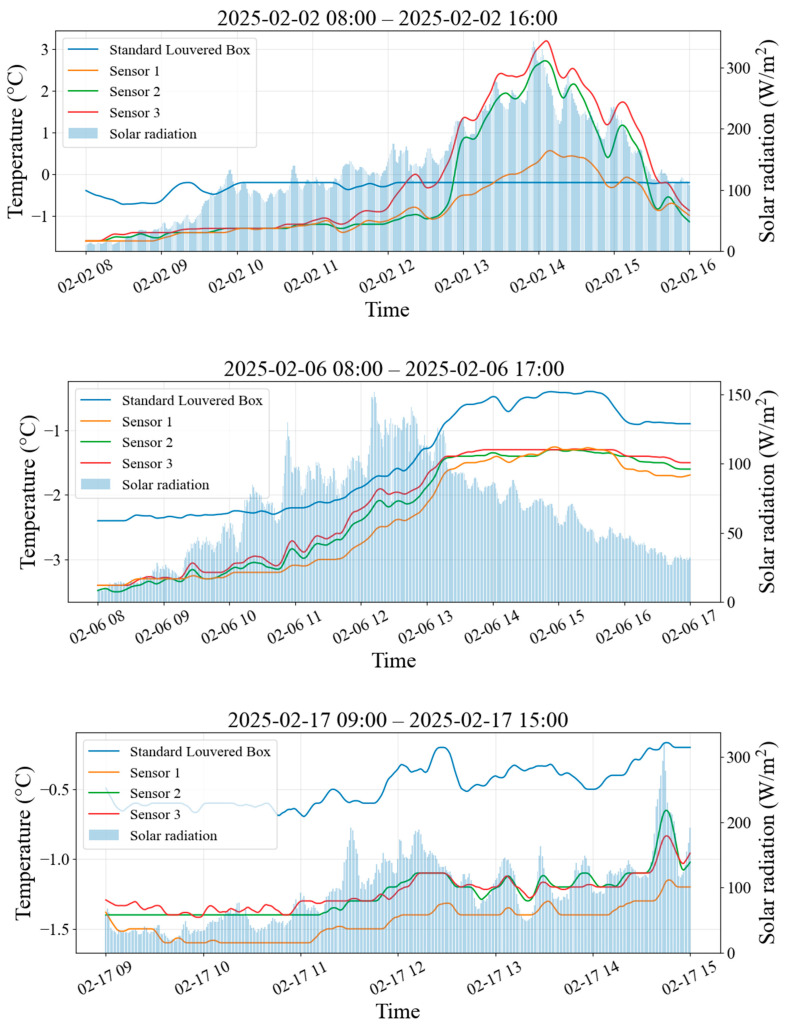
Time series of the Stevenson-screen reference temperature and the three field-deployed temperature sensors on the representative icing days (2 February, 6 February, and 17 February 2025). Panels correspond to three representative icing cases with different icing severities and radiative conditions.

This study uses 1 min resolution raw data from three representative observation days (2 February 2025, 6 February 2025, and 17 February 2025). The dataset includes the reference temperature in the Stevenson screen (Tref) and readings from three field-deployed temperature sensors (T1, T2, T3), corresponding to severe icing, mild icing, and nearly no icing conditions, respectively. Figure 9 presents the raw time series for these representative days, and solar radiation (R) was recorded synchronously.

**Figure 10:**
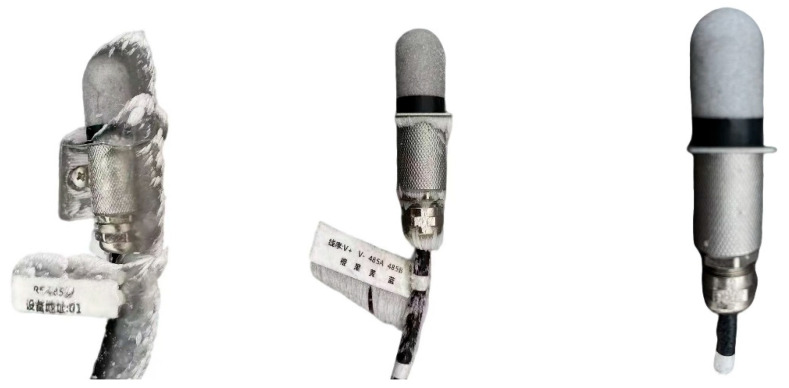
Field inspection photographs of Sensors 1, 2, and 3 during representative icing periods, illustrating the different icing severities under the three deployment configurations. The non-English text in the photograph is part of the original device label and does not affect the interpretation of the figure.

Icing severity was determined based on concurrent on-site inspection photographs (Figure 10).

Using the Stevenson-screen temperature as the reference, the instantaneous temperature deviation of the sensor is defined as:

(3) $$\Delta T_{i}(t)=T_{i}(t)-T_{ref}(t),i\in \{1,2,3\}$$

For each observation period, the mean deviation of $\Delta T_{i}$ ($\overset{-}{\Delta }T$) and the percentile interval (P5–P95) were computed to characterize the systematic bias and the primary range of variability, respectively. The bias statistics of the three sensors during the three representative icing periods are summarized in Table 4.

Ice accretion increases the effective thermal mass of temperature sensors, leading to a delayed response in temperature variations. To quantify this dynamic response difference, the cross-correlation peak method was used to estimate the response lag of Sensor 1 relative to Sensor 3 at an integer-minute resolution [38]. Considering that, under strong radiation, field-deployed probes may be additionally heated by shortwave radiation and thus exhibit temperature patterns that differ from the Stevenson-screen reference, Sensor 3 (with nearly no icing) was adopted as the field-deployed control. The lag was therefore computed between sensors of the same type to quantify the response difference attributable to icing.

A mild smoothing (5 min centered moving average) was first applied to the temperature time series to suppress short-term observational noise while preserving the minute-to-hour-scale evolution of the thermal response. The smoothing window was selected to avoid masking the turning points associated with icing and de-icing processes. A lag search window of −120 to +120 min was then adopted, which is substantially wider than the expected icing-induced response delays (typically on the order of several to tens of minutes) and therefore reduces the risk of truncating the true lag while avoiding spurious correlations caused by mismatches at much longer time scales. The shifted correlation coefficient was computed as follows [39]:

(4) $$r(\tau )=corr({\tilde{T}}_{1}(t),\;{\tilde{T}}_{3}(t-\tau ))$$

The shift that maximizes the correlation coefficient is taken as the optimal lag:

(5) $$\tau^{*}=arg\;\underset{\tau }{max}\;r(\tau )$$

When $\tau^{*}$ > 0, Sensor 1 is interpreted as lagging behind Sensor 3 by $\tau^{*}$ minutes. The maximum correlation coefficient ($r(\tau^{*})$) is also reported as an indicator of the reliability of the lag estimate. The response lags for the three representative icing periods are listed in Table 5.

To verify the robustness of the lag estimation, a sensitivity analysis was conducted by varying the smoothing window (3, 5, and 7 min) and the lag-search range (±60, ±120, and ±180 min). These settings were chosen to test whether the estimated lag was sensitive to reasonable changes in noise smoothing and search extent. Because 6 February showed the most pronounced lag, it was used as the representative case for detailed analysis. The corresponding results are summarized in Table 6.

As shown in Table 6, for the representative severe-icing case on 6 February, the estimated lag remained within a narrow range of 17–19 min under all tested parameter combinations, while the corresponding maximum correlation coefficient remained nearly constant (~0.984). Supplementary calculations further showed that the lag estimates for 2 February and 17 February remained unchanged at 5 min and 2 min, respectively, under alternative lag-search ranges. These results indicate that the lag estimation is robust and not sensitive to reasonable parameter choices.

On 6 February and 17 February, the severely iced Sensor 1 exhibited a stable cold bias relative to the Stevenson-screen reference, with mean deviations of −0.88 °C and −0.98 °C, respectively, both larger in magnitude than those of Sensors 2 and 3 under light or negligible icing. Cross-correlation analysis revealed a minute-scale delay of Sensor 1 relative to Sensor 3, with the most pronounced lag occurring on 6 February. On 2 February, the much smaller daytime positive bias of Sensor 1 relative to Sensors 2 and 3 further indicates that icing suppresses rapid shortwave-radiation-driven warming. Overall, ice accretion leads to both a cold bias and a delayed thermal response, with the most evident lag occurring during the severe-icing case on 6 February.

The observed lag behavior can be interpreted in terms of transient heat transfer [40]. A temperature sensor may be approximated as a first-order thermal system:

(6) $$\tau \frac{dT_{s}}{dt}+T_{s}=T_{a}+\frac{Q_{rad}}{H}\;$$

where $T_{s}$ is the sensor temperature, $T_{a}$ is the ambient air temperature, and τ is the effective thermal time constant, $Q_{rad}$ is the net radiative forcing acting on the sensor, and H is an effective heat-transfer coefficient. Under icing conditions, the observed lag can be interpreted as consistent with an increase in effective thermal mass and thermal resistance caused by ice accretion. As a result, more time is required for the sensor temperature to adjust to external thermal forcing, leading to a slower response to changes in air temperature and radiation. This mechanism explains why the severely iced Sensor 1 exhibited both a more pronounced cold bias and a delayed warming response relative to the lightly iced or nearly ice-free sensors. In particular, under daytime radiative forcing, the ice layer suppresses rapid shortwave-radiation-driven heating by increasing thermal inertia and weakening heat transfer efficiency, thereby producing a measurable minute-scale lag in the temperature response.

In a simplified physical picture, the response delay is expected to tend to increase with icing thickness because thicker ice generally implies both a larger effective thermal mass and a longer heat-transfer path between the ambient air and the sensing element. As the ice layer grows, more heat is required for the sensor assembly to adjust to external thermal forcing, and the efficiency of heat transfer from the surrounding air to the sensing element is further reduced. Consequently, the effective thermal time constant becomes larger, and the sensor response tends to be slower. However, under field conditions, this relationship may also depend on ice type, density, contact condition, radiation intensity, and wind speed, and therefore may not be strictly linear. Owing to the limited number of paired observations of icing thickness and response lag in the present dataset, no explicit parameterized model is established here. Instead, this thickness-dependent effect is discussed qualitatively and identified as an important direction for future work. Accordingly, no predictive model relating ice thickness to response time is proposed here, and the present interpretation should be regarded as preliminary because it is based on a limited number of representative icing events.

Another factor that may influence the unshielded Sensor 3 is the high reflectivity of surrounding snow- and ice-covered surfaces. In addition to direct solar radiation, Sensor 3 may also receive reflected shortwave radiation from nearby high-albedo surfaces, which could further increase the radiative load on the sensor body and housing and thereby enhance daytime warming bias. Because surface albedo and reflected shortwave radiation were not measured separately in the present field campaign, this contribution cannot be quantified explicitly. Nevertheless, this limitation mainly affects the detailed attribution of radiation-induced bias for the exposed configuration, rather than the broader conclusion that shielding, ventilation, and icing jointly influence temperature-measurement performance under natural icing conditions. Future studies should incorporate radiation-component measurements or local albedo observations to better separate the roles of direct and reflected radiative forcing.

### 3.3. The Impact of Icing on Cup Anemometers

**Figure 11:**
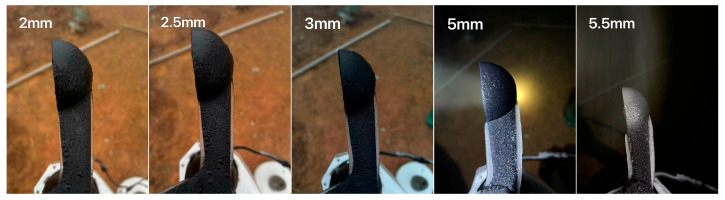
Field photographs showing the measured icing thickness on Cup 2 (2, 2.5, 3, 5, and 5.5 mm) during successive de-icing comparison events.

**Figure 12:**
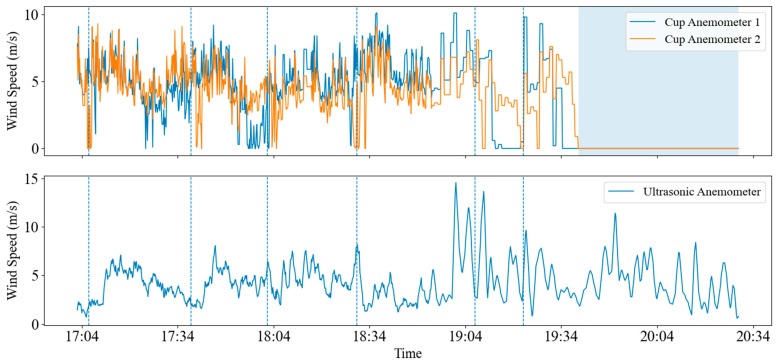
Raw wind-speed time series of Cup 1, Cup 2, and the co-located ultrasonic anemometer during the de-icing comparison experiment, showing the progressive divergence and eventual freezing-induced failure of Cup 2. The blue shaded region indicates the freezing-induced failure period.

Two cup anemometers were co-located and mounted at the same height for synchronous measurements: Cup 1 was manually de-iced, whereas Cup 2 was left untreated to allow natural ice accretion. An ultrasonic anemometer was additionally deployed to provide a synchronous wind-speed reference and to assist in identifying freezing-induced failure states. The dataset covers approximately 3 h with a sampling interval of 10 s. During this period, Cup 1 was manually de-iced multiple times at approximately 17:06, 17:38, 18:02, 18:30, and 19:22. At each de-icing time, the icing thickness on Cup 2 was measured simultaneously (Figure 11) as 2, 2.5, 3, 5, and 5.5 mm, respectively. Figure 12 presents the raw wind-speed time series over the three-hour period.

Continuous zero outputs from Cup 2 were identified as freezing-induced failure; data from these intervals were excluded from quantitative error analysis and used only to describe the failure phenomenon. Taking each de-icing event as the starting point, the period from 5 to 15 min after de-icing was selected as the “valid comparison window”. This treatment avoids transient disturbances immediately after de-icing, such as water shedding and short-term thermal effects, while ensuring that Cup 1 remains effectively ice-free and Cup 2 has resumed natural accretion under the same incoming flow.

The wind-speed ratio was defined as:

(7) $$Ratio=\frac{V_{cup2}}{V_{cup1}}$$

and the wind-speed underestimation percentage was then calculated as:

(8) B=(1−R)×100%

As shown in Figure 12, the two cup anemometers produced generally consistent outputs during the initial stage of the experiment. After successive de-icing events, however, their responses gradually diverged: Cup 1 continued to respond rapidly to variations in incoming wind, whereas Cup 2 reported persistently lower wind speed under the same flow and eventually entered a freezing-induced failure stage characterized by continuous zero outputs.

Table 7 shows that when the icing thickness was 2 mm, Cup 2 exhibited only slight underestimation relative to Cup 1 (R = 0.946, ~5.4%). As the ice thickness increases from 2 mm to 2.5–3 mm, the underestimation rises markedly (R decreases to 0.810–0.780, corresponding to ~19–22%). With further accretion to 5–5.5 mm, the underestimation remains at ~15–21% (R = 0.846 and 0.791), indicating that the deterioration trend was clearly nonlinear rather than strictly monotonic. This behavior suggests that wind-speed underestimation generally intensified with icing development, but the incremental effect weakened once the cup anemometer approached a severely degraded rotational state, indicating a saturation-like tendency.

To conceptually link icing thickness with wind-speed measurement degradation, a simple empirical relationship may be written as *B* = f(h), where *B* denotes the underestimation percentage and h is the icing thickness. Based on the present field observations, thicker accretion generally corresponded to stronger underestimation, indicating a thickness-dependent deterioration trend. However, the relationship was not strictly monotonic and showed substantial scatter, suggesting that icing thickness alone cannot fully determine the error magnitude. In practice, the observed underestimation is also affected by icing morphology, blockage of the cup opening, surface roughness, uneven mass distribution, and time-varying mechanical resistance. Therefore, the present dataset supports only a qualitative or semi-quantitative empirical interpretation of the thickness–error relationship, rather than a predictive semi-empirical model. The establishment of a more robust model that jointly incorporates icing thickness, morphology, and flow conditions remains an important direction for future work. Accordingly, the present results should be interpreted primarily as strong field evidence for an icing-related deterioration tendency, rather than as a generally established quantitative model applicable across different icing conditions.

From a mechanistic perspective, icing affects cup anemometers through two coupled pathways. The first is aerodynamic degradation: ice accretion can alter the effective geometry and drag characteristics of the cup surfaces, which likely reduces the aerodynamic driving torque and contributes to underestimation. The second is enhanced mechanical impediment: icing accretion increases rotor inertia and may also increase shaft friction, making start-up more difficult in low-wind regimes and attenuating the response to gusts; in extreme cases, this leads to sticking and ultimately freezing-induced failure. These geometric and surface changes may also alter the effective aerodynamic characteristics of the rotating cups, although a quantitative Reynolds-number-based analysis is beyond the scope of the present field dataset. Combined with the continuous zero outputs of Cup 2 in the later part of Figure 12, it can be concluded that the instrument had entered a “freezing-failure” state. At this point, the anemometer no longer provides valid wind-speed measurements.

The present field observations are broadly consistent with previous wind-tunnel and laboratory studies, which have shown that ice accretion on cup anemometers reduces rotational efficiency, increases wind-speed underestimation, and may eventually lead to stall or complete failure under severe icing. Compared with controlled laboratory conditions, however, the field results in this study exhibit greater variability and weaker monotonicity, likely because natural icing involves nonuniform accretion, evolving ice morphology, fluctuating flow conditions, and intermittent melting–refreezing processes. From an operational perspective, these findings indicate that wind-speed quality control in icing environments should include an automatic flagging rule for persistent near-zero outputs with ceased response, so that failed data are excluded from icing prediction, de-icing decision-making, or other wind-dependent monitoring applications.

## 4. Conclusions

This study investigated the effects of natural icing on field measurements of air temperature and wind speed. The results show that measurement degradation in icing environments is governed by the combined effects of radiative forcing, installation conditions, and icing state. For temperature measurements, solar radiation was identified as the dominant external driver of daytime positive bias, whereas shielding and ventilation determined the magnitude and variability of measurement error. Under icing conditions, temperature sensors exhibited both systematic cold bias and delayed thermal response. For wind-speed measurements, natural icing caused underestimation in cup anemometers and, under severe accretion, eventually led to freezing-induced failure.

From a practical perspective, the findings provide direct guidance for power meteorological monitoring in icing-prone regions. Temperature probes deployed in the field should not be regarded as equivalent to standardized reference measurements unless adequate shielding and ventilation are ensured. For cup anemometers, persistent near-zero output during icing events should be treated as a likely indicator of freezing-induced failure rather than valid low-wind conditions, and corresponding quality-control procedures should be incorporated into operational monitoring systems. Redundant or complementary measurements may further improve data reliability at key monitoring sites under severe icing conditions.

The broader significance of this study lies in its field-based identification of sensor error mechanisms and degradation patterns under natural icing. These findings are potentially applicable to other cold, humid, and icing-prone outdoor environments, especially in complex terrain where radiation, airflow, and icing interact strongly. However, the specific numerical values reported here should not be directly transferred to all regions or sensor types, because they remain dependent on local meteorological conditions, terrain, icing morphology, and installation details. Further validation under more sites and environmental regimes is therefore needed.

Future work should therefore focus on three aspects: first, multi-site and multi-event validation under different terrain and icing regimes; second, improved characterization of icing morphology, radiative environment, and flow conditions so that more robust semi-empirical or physically informed models can be developed; and third, optimization of sensor shielding, ventilation, anti-icing or de-icing strategies, and automated quality-control rules for reliable operational use in severe icing environments.

## Figures and Tables

**Table 1:** Sensor deployment configurations.

Environmental Condition	Sensor ID	Installation Method	Features
	1	Miniature Stevenson screen	Typical power-grid micro-meteorological installation; ventilated (with convection); no radiation exposure
Winter low temperature (non-icing)	2	Transparent glass cover	No convection; radiation exposure
	3	No additional shielding	With convection; radiation exposure
	1	Windward side	Severe icing
Icing period	2	Leeward side	Mild icing
	3	Under polymer cover	Basically no icing

**Table 2:** Specifications of the sensors used in this study.

Sensor	Model	Accuracy	Resolution	Response Time
Temperature–humidity sensor	RS-WS-N01	±0.5 °C/±4%RH	0.1 °C/0.1%RH	≤25 s/≤8 s
Cup anemometer	RS-FSA-N01	±(0.2 + 0.03 V) m/s	0.1 m/s	≤2 s
Ultrasonic anemometer	RS-FSXCS	±(0.2 ± 0.02 V) m/s	0.01 m/s	≤1 s

Note: The temperature response time is defined as the time constant of a first-order system (τ63); V is the true wind speed.

**Table 3:** Linear regression results for the relationship between temperature error and solar radiation under three sensor configurations.

Sensor	Intercept (°C)	Slope (°C·(W·m^−2^)^−1^)	95% CI of Slope	*p*-Value	Error Increase per 100 W/m^2^ (°C)	R^2^
Sensor 1	−1.0783	0.001511	[0.001460, 0.001561]	<0.001	0.1511	0.4039
Sensor 2	−1.4832	0.008597	[0.008499, 0.008694]	<0.001	0.8597	0.8760
Sensor 3	−1.2842	0.004103	[0.003985, 0.004220]	<0.001	0.4103	0.5712

**Table 4:** Bias statistics of the three sensors relative to the Stevenson-screen reference across the three periods.

Time Interval	$\overset{-}{\Delta }T$1	$\overset{-}{\Delta }T$2	$\overset{-}{\Delta }T$3	Sensor 1 P5–P95 (°C)	Sensor 2 P5–P95 (°C)	Sensor 3 P5–P95 (°C)
2 February 2025 08:00–16:00	−0.57	−0.08	+0.24	[−1.20, +0.60]	[−1.10, +2.40]	[−1.10, +2.80]
6 February 2025 08:00–17:00	−0.88	−0.78	−0.69	[−1.00, −0.70]	[−1.10, −0.50]	[−1.00, −0.30]
17 February 2025 09:00–15:00	−0.98	−0.78	−0.77	[−1.10, −0.90]	[−0.90, −0.60]	[−0.90, −0.60]

**Table 5:** Response lag of Sensor 1 relative to Sensor 3 during the three representative icing periods.

Time Interval	$\tau^{*}$ (min)	$r(\tau^{*})$
2 February 2025 08:00–16:00	5	0.982
6 February 2025 08:00–17:00	18	0.984
17 February 2025 09:00–15:00	2	0.936

**Table 6:** Sensitivity of lag estimation to smoothing and lag-search settings for the representative severe-icing case on 6 February.

Case	Smoothing Window (min)	Lag Search Window (min)	$\tau^{*}$ (min)	$r(\tau^{*})$
6 February 2025 08:00–17:00	3	±60	17	0.9839
	3	±120	17	0.9839
	3	±180	17	0.9839
	5	±60	18	0.9844
	5	±120	18	0.9844
	5	±180	18	0.9844
	7	±60	19	0.9849
	7	±120	19	0.9849
	7	±180	19	0.9849

**Table 7:** Wind-speed underestimation percentage corresponding to icing thickness.

Time Interval	Sample Size	Cup 2/Cup 1	Underestimation (%)	Icing Thickness (min)
17:11–17:21	55	0.946	5.4%	2
17:43–17:53	50	0.810	19%	2.5
18:07–18:17	51	0.78	22%	3
18:35–18:45	36	0.846	15.4%	5
19:22–19:37	31	0.791	20.9%	5.5

## Data Availability

The data supporting the findings of this study are available from the corresponding author upon reasonable request.
